# Concurrent Cemento-Osseous Dysplasia and Osteogenic Sarcoma: Report of Two Cases

**DOI:** 10.1155/2012/180561

**Published:** 2012-03-11

**Authors:** A. A. Olusanya, B. F. Adeyemi, A. O. Adisa

**Affiliations:** ^1^Department of Oral and Maxillofacial Surgery, University College Hospital, Ibadan, Nigeria; ^2^Department of Oral Pathology/Oral Medicine, University College Hospital, Ibadan, Nigeria

## Abstract

*Aim*. Cemento-osseous dysplasia (COD) represents a rare group of benign fibroosseous disorders, while osteogenic sarcoma (OS) on the hand, is a malignant tumour of ominous prognosis. A combination of COD and OS is rare and sparsely reported in literature. There are only four reported cases known to the authors. The aim of this paper is to report additional cases of COD occurring concurrently with OS. *Materials and Methods*. The clinicoradiologic findings and histological analysis of mandibular lesions in two patients who reported at the Dental Centre of the University College Hospital, Ibadan, Nigeria, are presented. *Results*. The two patients were diagnosed of mandible osteosarcoma occurring concurrently with bilateral mandibular focal cemento-osseous dysplasia. *Conclusion*. The simultaneous occurrence of osteosarcoma and cemento-osseous dysplasia raises the question of whether COD has transformed into OS or a collision tumour has occurred and their simultaneous occurrence is just a coincidence.

## 1. Introduction

Cemento-osseous dysplasia (COD) represents a rare group of benign fibroosseous disorders in which bone is resorbed and replaced with a cementum-like material in a background of fibrous stroma [1, 2]. It is frequently asymptomatic and usually discovered incidentally on radiographs. Osteogenic sarcoma (OS), on the, hand, is a malignant tumour of ominous prognosis with symptoms such as pain and swelling. A combination of COD and OS is rare and therefore sparsely reported in literature. There are only four reported cases known to the authors. The aim of this paper is to report two additional cases of COD occurring concurrently with OS.

## 2. Case  1

**Figure 1:**
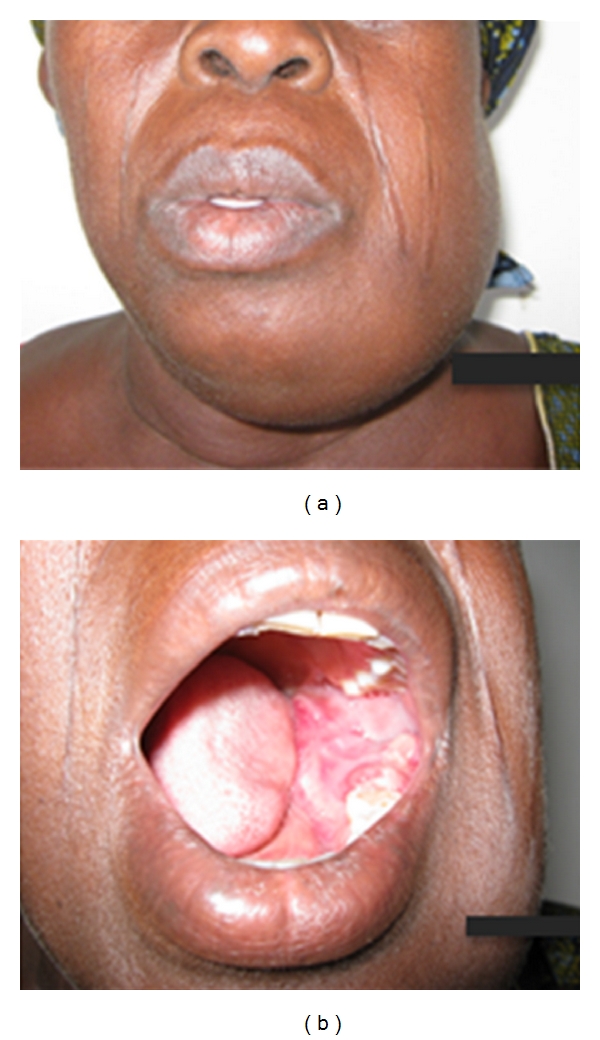
Clinical picture of case 1.

**Figure 2:**
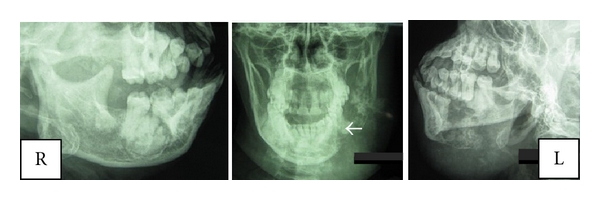
Oblique lateral views of the mandible and posteroanterior view of the jaws for case 1 demonstrating an osteolytic lesion (with centrifugal radiopaque spikes) within which the faint presence of an irregular, shapeless mass of radiopacity can be seen–white arrow.

**Figure 3:**
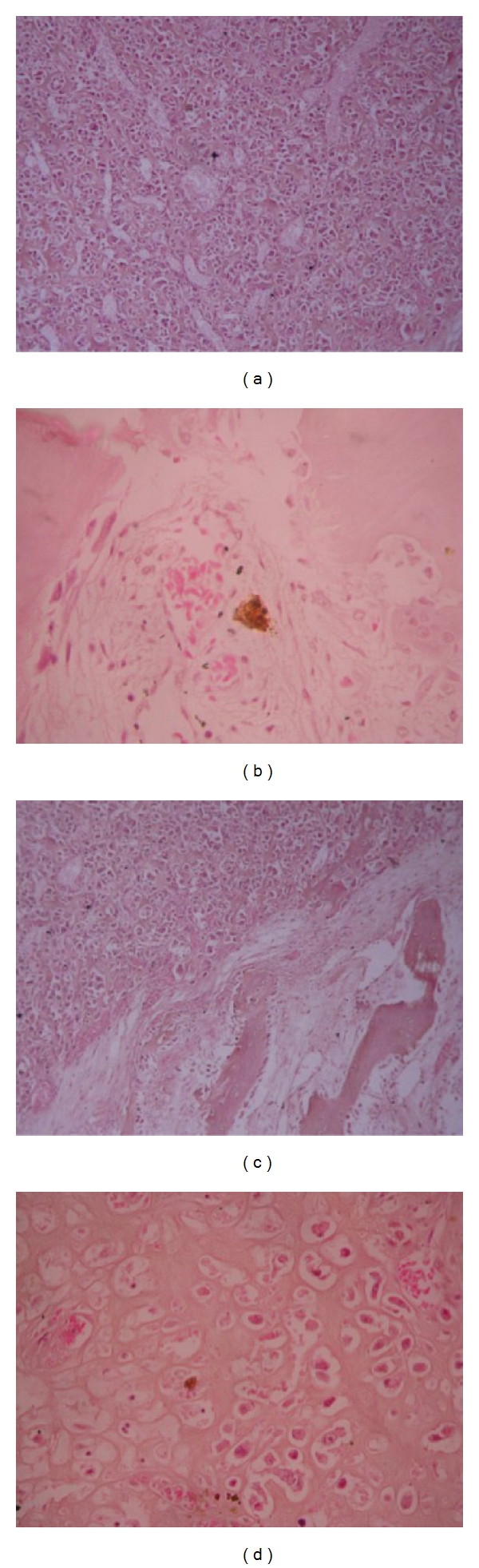
(a) Osteoblastic variant of osteogenic sarcoma of case  1 showing osteoblasts with cytonuclear pleomorphism and hyperchromatic nuclei surrounding malignant osteoid (X100). (b) Cemento-osseous dysplasia showing a benign mesenchymal neoplasm composed of irregular islands of immature bone with osteoblastic rimming surrounded by a moderately dense fibrous connective tissue stroma (X100). (c) A possible transition zone between osteogenic sarcoma and cemento-osseous dysplasia (X400). (d) Chondroblastic osteosarcoma of case 2 showing a malignant mesenchymal neoplasm consisting of pleomorphic chondroblast with prominent hyperchromatic nuclei amidst malignant osteoid(X100).

A 65-year-old Nigerian female reported at the oral and maxillofacial surgery clinic of Dental Centre, University College Hospital, Ibadan, Nigeria, with a six-month history of a left-sided mandibular swelling which was of spontaneous origin and painful intermittently. The swelling extended from the left ramus to the left parasymphyseal region of the mandible. The overlying skin was intact, appeared clinically normal, and not attached to the underlying tumour. The associated teeth were mobile, and both the lingual and buccal sulci were obliterated by the tumour mass (Figure 1). There was neither obvious clinical swelling nor pathology on the right side of the mandible. The radiographic appearance is as shown in Figure 2. The left lateral oblique view of the mandible and left side of the posteroanterior jaw radiograph demonstrated an osteolytic lesion (with centrifugal radiopaque spikes) within which the faint presence of an irregular, shapeless mass of radiopacity can be seen (white arrow). The right lateral oblique view of the mandible showed a distinct, irregular, lobulated mass situated apically between lower 16 and 17 which extended to the cortical margin of the lower border of the mandible. The lesion was limited peripherally by a thin band of radiolucency. An incisional biopsy of the left mandibular mass revealed an osteogenic sarcoma (Figure 3). A workup for surgery was then carried out including investigation for possible distant metastasis. Metastatic workup such as chest radiograph and abdominopelvic ultrasound was negative as these did not reveal any metastatic lesions. Patient had a left hemimandibulectomy procedure done as well as the surgical excision of the fibroosseous lesion on the right. Histological analysis of the surgical specimens confirmed an osteogenic sarcoma on the left side of the jaw and a cemento-osseous dysplasia on the right side of the jaw (Figure 3).

## 3. Case  2

**Figure 4:**
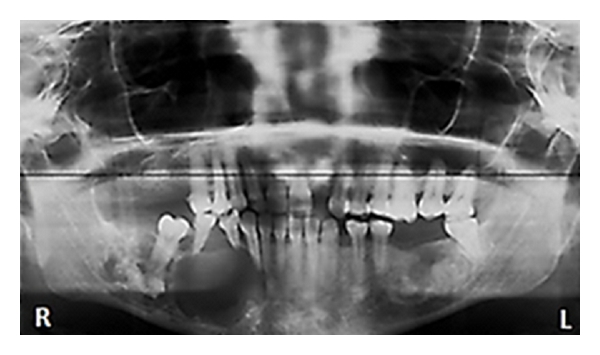
An orthopantomogram of the patient in case 2 showing a significant bone destruction of the right mandible extending from the lower right lateral incisor to a region just short of the mandibular angle. The radiolucency also encompasses the irregular mass of radio-opacity just apical to right mandibular first molar. The radiograph shows a similar irregularly shaped radio-opacity within the body of the left mandible.

A 45-year-old Nigerian female reported at our clinic with a 3-month history of a right mandibular swelling of spontaneous origin which was otherwise not symptomatic but was increasing progressively in size. An orthopantomogram revealed a significant bone destruction of the right mandible extending from the lower right lateral incisor to a region just short of the mandibular angle. The radiolucency also encompasses the irregular mass of radio-opacity just apical to 16. The radiograph shows a similar irregularly shaped radio-opacity within the body of the left mandible (Figure 4). An incisional biopsy of the right mandibular lesion revealed chondroblastic osteogenic sarcoma (Figure 3(d)). When the patient was counselled about the malignant nature of her disease, she opted for a traditional mode of treatment and efforts to convince her otherwise failed. She was lost to followup.

## 4. Discussion

COD is a nonneoplastic fibroosseous lesion that is believed to run a relatively benign course. It is commonly asymptomatic and usually discovered on routine radiographs. This lesion typically affects middle-aged women of African and Asian descent, but it can occur in Caucasians as well [2]. COD has been categorised into three subtypes based on their extent and radiographic appearance; periapical, solitary/focal and florid/multiple [2, 3]. The periapical subtype presents as discrete masses of varying radiopacities at the apices of teeth, especially the mandibular anterior teeth which typically remain vital. The focal subtype is typically a solitary, often lobulated lesion presenting bilaterally, while the florid subtype presents as irregular, multiple masses of mixed radiopacities occurring in more than two quadrants of the jaw. These subtypes are histologically similar presenting as a mixture of bone and cementum-like material on a background of fibrous stroma. It is also worthy of note to bear in mind that the histological differentials of COD include fibrous dysplasia, ossifying fibroma, chronic osteomyelitis, and Paget's disease [2]. These lesions should therefore be differentiated from COD by making summative assessment to include both history of the lesion and clinical findings. The management of COD is conservative unless when symptomatic as a result of superimposed infection as COD is avascular and therefore susceptible to infection [1, 4]. Therefore, when the lesion becomes exposed into the oral cavity for example following tooth extraction, surgical biopsy or erosion of the overlying mucosa infection can occur [1, 2]. Osteomyioelitis resulting from infected lesions is difficult to treat as there is widespread sclerosis with consequent poor blood supply. In some rare situations where COD has caused an aesthetically unpleasant swelling, a surgical intervention may be required [4]. COD has been associated with some lesions such as solitary bone cyst, aneurysmal bone cyst [3], and chronic osteomyelitis [4].

Osteogenic sarcoma (OS) is a highly malignant tumour of bone that is associated with a poor prognosis. Its aetiology is unknown but a few cases have been known to occur in irradiated or previously diseased bone [4, 5]. OS of the jaws is uncommon and accounts for about 5% to 10% of all OSs [5, 6]. This tumour is typically seen in the third and fourth decades of life (a decade later than the osteosarcoma of long bones), commoner among the male gender and classically presents with a rapidly growing swelling which is commoner in the mandible than the maxilla and is usually painful [4, 5]. Radiographic appearances are variable, but essentially bone destruction predominates over bone formation giving varied poorly defined radiolucencies which have been described as “sunray appearance,” “sunburst appearance,” “moth eaten appearance,” “Codman's triangle.” A chest radiograph may reveal metastasis as some of these lesions metastasize early. Histologically, islands of immature bone are seen dispersed within a background of richly vascularised fibrous connective tissue stroma. The treatment of OS is radical and includes both surgical excision (with anatomic barrier sacrifice) combined with chemotherapy and/or radiotherapy [5]. Prognosis is generally poor but may be dependent on the extent of the lesion at the time of treatment; lesions less than 5 cm have 25% to 40% five-year survival rate, while lesions more than 15 cm have almost zero percent five year survival rate [4, 5]. OS has been known to a risein a number of other lesions like Paget's disease, chronic osteomyelitis and central giant cell granuloma, fibrous dysplasia, multiple osteochondromas, bone infarct, and osteogenesis imperfecta [5].

COD and OS are two independent disease processes that run entirely different courses with varying treatment options and differing prognosis. These two distinctively different lesions occurring simultaneously in the same jaw are a rarity and therefore sparsely reported in literature. There are only four reported cases of a sarcoma occurring simultaneously with COD known to the authors.

Schneider et al., in 1999, reported a case of a 54-year-old black woman seen in 1979 with mandibular “malignant spindle cell tumour” occurring in florid osseous dysplasia [7]. The tumour was diagnosed histologically as malignant fibrous histiocytoma, but immunohistochemistry was not available at the time to confirm this diagnosis. In their report, a transition zone between these two lesions was cited though they could not rule out the possibility of a collision phenomenon. Their case represents the first reported malignancy occurring with a cement-osseous dysplasia.

Cheng et al., in 2002, reported a case of a 72-year-old black female with a week history of left mandibular swelling and numbness of the lower lip [6]. The patient had been diagnosed of Paget's disease of bone three years earlier. Imaging revealed an osteolytic lesion of the left body of the mandible as well as generalised, diffuse, patchy, cotton wool-like radio-opacity which was subsequently diagnosed as florid cemento-osseous dysplasia (FCOD) adjacent to pagetic bone, while the left mandibular swelling was diagnosed as osteosarcoma. In their discussion, they stated that though there are many similarities between FCOD and Paget's disease, it is generally believed that FCOD does not have the potential for malignant transformation. They further went on to conclude that the osteosarcoma arose from the Pagetoid bone and not the FCOD as the OS was found in the centre of Pagetic bone, not in the area of florid cemento-osseous dysplasia [6]. Nevertheless, their report represents one of the reported cases in literature of OS occurring concurrently with COD as earlier cited by Melrose and Handlers [8].

Melrose and Handlers in 2003 reported a case of a 36-year-old black woman with an increasing, painful, mandibular swelling who had radiographic evidence of FCOD as a bignathic presentation on previous radiographs taken three years before presentation at their hospital [8]. The biopsy of the mandibular swelling was stated as high-grade osteosarcoma, while a core biopsy of the lesion on the contralateral side of the mandible revealed features in accordance with cemento-osseous dysplasia. The patient subsequently had mandibular resection, but was lost to follow up after discharge. Though the authors stated their report as the second one reported in literature citing a description of the case of Cheng et al. as the first, Schneider's report was three years earlier than Cheng's making it the first reported case of simultaneous occurrence of a sarcoma and a cemento-osseous dysplasia [7].

Lopes et al, in 2010, reported a similar case to that of Melrose and Handlersin a clinic-pathologic conference as a 44-year-old black female who presented with a painful swelling of the right mandible which was diagnosed as osteosarcoma in a background of FCOD [9].

All the above previous cases have been associated with the florid subtype of cemento-osseous dysplasia which is said to be similar to Paget' disease of bone except that FCOD is localized to the jaws in the tooth-bearing portion. Based on this comparability, FCOD has been referred to as the “Paget 's disease of the mandible” [6, 9]. The present cases in this paper' however, are the first known to the author as OS associated with the focal subtype of COD, and they may represent the fifth and sixth cases of OS occurring concurrently with COD reported in literature.

## 5. Conclusion

The simultaneous occurrence of osteosarcoma and cemento-osseous dysplasia, though rare, raises the question of whether a transition can occur from COD to OS or these cases simply represent collision tumours and their simultaneous occurrence is just a coincidence. The “transition zone” observed in this paper is not to state that a definite evidence of OS arising from COD has been found but that the two lesions are in close proximity with each other even microscopically. Further investigation will therefore be required to establish a transition, if any exists, from COD to OS. Nevertheless, patients with COD especially the florid and focal subtypes may benefit from long-term followup.

## References

[1] Singer SR, Mupparapu M, Rinaggio J (2005). Florid cementoosseous dysplasia and chronic diffuse osteomyelitis: report of a simultaneous presentation and review of the literature. *Journal of the American Dental Association*.

[2] Dagistan S, Tozoglu U, Goregen M, Cakur B (2007). Florid cement-osseous dysplasia: a case report. *Medicina Oral, Patología Oral y Cirugía Bucal*.

[3] Mahomed F, Altini M, Meer S, Coleman H (2005). Cemento-osseous dysplasia with associated simple bone cysts. *Journal of Oral and Maxillofacial Surgery*.

[4] Cawson RA (2002). Chapter 8 Odontogenic tumours and tumour like lesions of the jaws. *Essentials of Oral Pathology and Oral Medicine*.

[5] Regezi JA, Sciubba JJ, Jordan RCK (2003). Oral pathology. *Clinical Pathologic Correlations*.

[6] Cheng YSL, Wright JM, Walstad WR, Finn MD (2002). Osteosarcoma arising in Paget’s disease of the mandible. *Oral Oncology*.

[7] Schneider LC, Dolinsky HB, Grodjesk JE, Mesa ML, Doyle JL (1999). Malignant spindle cell tumor arising in the mandible of a patient with florid osseous dysplasia. *Oral Surgery, Oral Medicine, Oral Pathology, Oral Radiology, and Endodontics*.

[8] Melrose R, Handlers J (2003). Osteosarcoma ex florid osseous dysplasia: report of a case. *Oral Surgery, Oral Medicine, Oral Pathology, Oral Radiology, and Endodontology*.

[9] Lopes MA, Kim HS, Mariano FV, Corrêa MB, de Rabelo NTA, Vargas PA (2010). Clinico-pathologic conference: case 1. *Head and Neck Pathology*.

